# Application and trend of bioluminescence imaging in metabolic syndrome research

**DOI:** 10.3389/fchem.2022.1113546

**Published:** 2023-01-09

**Authors:** Shirui Li, Kang Wang, Zeyu Wang, Wenjie Zhang, Zenglin Liu, Yugang Cheng, Jiankang Zhu, Mingwei Zhong, Sanyuan Hu, Yun Zhang

**Affiliations:** ^1^ Department of General Surgery, Shandong Provincial Qianfoshan Hospital, Shandong University, Jinan, China; ^2^ Department of General Surgery, The First Affiliated Hospital of Shandong First Medical University, Jinan, China; ^3^ Postgraduate Department, Shandong First Medical University, Jinan, China

**Keywords:** adipose inflammation, bioluminescence imaging, circadian rhythm disorders, metabolic syndrome, model, non-alcoholic fatty liver, obesity, type 2 diabetes

## Abstract

Bioluminescence imaging is a non-invasive technology used to visualize physiological processes in animals and is useful for studying the dynamics of metabolic syndrome. Metabolic syndrome is a broad spectrum of diseases which are rapidly increasing in prevalence, and is closely associated with obesity, type 2 diabetes, nonalcoholic fatty liver disease, and circadian rhythm disorder. To better serve metabolic syndrome research, researchers have established a variety of animal models expressing luciferase, while also committing to finding more suitable luciferase promoters and developing more efficient luciferase-luciferin systems. In this review, we systematically summarize the applications of different models for bioluminescence imaging in the study of metabolic syndrome.

## 1 Introduction

Metabolic syndrome (MetS) is a systemic metabolic disorder associated with insulin resistance, obesity, dyslipidemia, and hypertension (Grundy et al., 2005). It also increases the risk of type 2 diabetes, non-alcoholic fatty liver disease (NAFLD), and cardiovascular disease (Eckel et al., 2005). These complications result in MetS being strongly associated with increased cardiovascular outcomes and overall mortality (Mottillo et al., 2010). Over the past few decades, the prevalence of MetS has dramatically increased. According to data from the National Health and Nutrition Examination Survey (NHANES) of the United States, an epidemiological survey involving 17,048 people from 2011 to 2016 showed that the prevalence of MetS in adults was 34.7%. MetS also shows a clear trend of youth. The prevalence among people aged 20–39 years increased significantly from 2015 to 2016 compared to that in 2011–2012 (from 16.2% to 21.3%) (Hirode and Wong, 2020). Given the rapidly increasing prevalence of MetS, researchers are becoming increasingly interested in its pathogenesis and potential treatment strategies. MetS is recognized as a systemic and dynamic process; therefore, its pathogenesis and response to therapeutic measures *in vivo* requires further investigation. Current epidemiological, clinical, and experimental studies highlight the importance of observing the pathological processes of MetS *in vivo* (Han et al., 2010).

**FIGURE 1:**
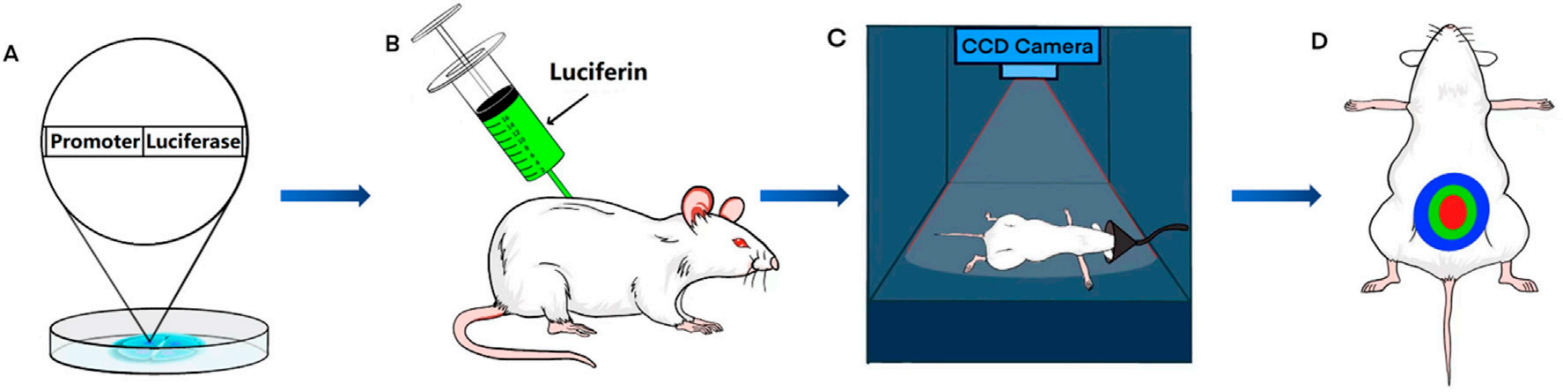
The process of bioluminescence imaging (BLI) in a mouse model. **(A)** Using transgenic technology, embryos are made to contain the luciferase gene under the control of a specific promoter. **(B)** Transgenic mice are injected intraperitoneally with fluorescein. **(C)** After anesthetizing the transgenic mice, a charge-coupled device (CCD) camera is used for signal acquisition. **(D)** Bioluminescence imaging is obtained from transgenic mice.

Bioluminescence imaging (BLI) is a technique used for visualizing physiological processes in animals. This technology is based on the research and application of bioluminescence, a natural phenomenon in which living organisms produce fluorescence (Badr and Tannous, 2011). Researchers have discovered the mechanism by which luciferase reacts with luciferin to generate light and have developed BLI (Mezzanotte et al., 2017). In animal research, standard techniques, such as immunohistochemistry and Western blot analysis, necessitate the euthanasia of an animal to analyze the physiopathological condition at a specific time point. In contrast, BLI can image animals without euthanasia and multiple times to evaluate changes in physiological processes over time. Since luciferase is not expressed in most animals, transgenic animal models are created by modifying their DNA with specific luciferase genes (Contag et al., 1997). Following the injection of luciferin as a substrate, luciferase in transgenic animals interacts with luciferin to produce light of a specific wavelength. Researchers use charge-coupled device (CCD) cameras to visualize the physiological processes of transgenic animals in a non-invasive manner (Zhang et al., 1999). This technology provides unprecedented assistance in the study of MetS (Figure 1).

The practicability of BLI has advanced significantly over the last decade (Luker and Luker, 2010; Alsawaftah et al., 2021). In this review, we focused on the application of BLI in the field of MetS and emphasized the role of BLI animal models in MetS-related diseases such as type 2 diabetes, NAFLD, and circadian rhythm disorder.

## 2 Application of BLI in type 2 diabetes

The pathophysiology of type 2 diabetes generally includes peripheral insulin resistance and pancreatic islet dysfunction. Insufficient insulin secretion from β-cells leads to pancreatic islet dysfunction. Decreased β-cell number and function are direct causes of decreased insulin secretion (Mizukami and Kudoh, 2022). Although histochemical methods can accurately determine the number and functional status of pancreatic β-cells in mice, it is impossible to repeatedly monitor their dynamic changes in one animal. BLI is an effective tool for monitoring changes in pancreatic β-cell number and function during diabetes progression in animal models (Kang et al., 2019).

BLI has been used to study diabetes for over 20 years (Table 1). Previous studies have confirmed that the *Mouse insulin I gene* is mainly expressed in pancreatic islet β-cells (Deltour et al., 1993). Park et al. constructed transgenic mice (MIP-Luc mice) expressing firefly luciferase under the control of *mouse insulin promoter* (MIP). This study used the Xenogen IVIS 200 imaging system to perform BLI of anesthetized MIP-Luc mice. A strong fluorescent signal was detected in the pancreatic region of mice injected intraperitoneally with D-luciferin. Western blot analysis and immunohistochemical studies showed that luciferase was only expressed in islet β-cells and was not detected in other tissues, including other cell components of the islet. The results of the insulin glucose tolerance test in MIP-Luc and wild-type mice were similar, which proved that the knock-in of the luciferase gene did not significantly change the physiological characteristics of the mice. There was a significant positive correlation between bioluminescence intensity and the number of islets. *In vitro* BLI of different numbers of isolated islets from MIP-Luc mice revealed that bioluminescence signal intensity and luciferase activity were positively correlated with islet number (Park et al., 2005). However, how to determine whether the bioluminescence signal intensity accurately reflects the changes in the number of β cells rather than the changes in the transcriptional regulation level of the luciferase promoter poses a problem. In 2009, Park et al. investigated the correlation between bioluminescence signals and β-cell numbers in MIP-Luc mice fed a normal or high-fat diet (Park and Bell, 2009). They found that β-cell mass and bioluminescence signal intensity increased with age and were more pronounced in mice fed a high-fat diet. In high-fat-fed MIP-Luc mice, there was a strong correlation between fasting insulin levels, β cell numbers, and bioluminescence signals. The MIP-Luc mouse model provides an accurate and non-invasive method for studying changes in β-cell number and function. Virostko et al. also constructed a transgenic mouse model (MIP-Luc-Vu mice) using MIP. After the injection of streptozotocin (STZ) into MIP-Luc-Vu mice, the bioluminescence signal intensity continued to decrease. Morphometric analysis also showed a proportional reduction in the number of luciferase-expressing β cells after STZ treatment. Bioluminescence was observed for more than 1 year after a certain number of islets from MIP-Luc-VU mice were transplanted into wild-type mice under the renal capsule or into the liver through the portal vein. The bioluminescence intensity at both the transplant sites showed a linear correlation with the number of islets. However, researchers also found that BLI was more sensitive in measuring islets under the renal capsule than in the liver, suggesting that tissue thickness absorbs more photons, which affects the detection of bioluminescence intensity. This result reveals the need to develop new luciferins with enhanced tissue penetration. Developing more efficient algorithms may also compensate for the effect of tissue thickness on BLI (Virostko et al., 2010). Sekiguchi et al. used male MIP-Luc-VU mice to mate with female wild-type mice, successfully detected the bioluminescence signal from embryos in the uterine cavity of pregnant wild-type mice by BLI, and determined the process of fetal β-cell regeneration (Sekiguchi et al., 2012). Previous studies have demonstrated that mouse embryos develop fully differentiated β-cells on day 13 (Sander et al., 2000). However, researchers have only been able to detect bioluminescence signals after 16 days. This may be due to an insufficient number of β-cells or low level of gene transcription in the early stage. Therefore, technological innovations are required for the detection of weaker bioluminescence signals. Yin et al. used an MIP-Luc mouse model to study endogenous β-cell regeneration. They found that pioglitazone combined with alogliptin enhanced β cell regeneration, which promoted the progress of β cell regeneration therapy in patients with diabetes (Yin et al., 2013).

**TABLE 1:** Promoters and luciferase-luciferin systems commonly used for BLI in metabolic syndrome research.

Promoter	Luciferase	Luciferin	References
Type 2 diabetes			
*Mouse insulin I promoter*	Firefly luciferase	D-luciferin	Marcheva et al., 2010, Yin et al., 2013, Virostko et al., 2010, Sekiguchi et al., 2012, Patel et al., 2014
*Rat insulin promoter*	Firefly luciferase	D-luciferin	Smith et al. (2006)
	RLuc-YFP fusion protein	Coelenterazine	Ghislain et al. (2016)
*Bmp4 promoter*	Firefly luciferase	D-luciferin	Yasunaga et al. (2011)
*Insulin I gene promoter*	Firefly luciferase	D-luciferin	Katsumata et al. (2013)
Circadian rhythm disorder			
*Mouse Per1 promoter*	Firefly luciferase	D-luciferin	Yamazaki et al. (2000)
*Mouse Per2 promoter*	Firefly luciferase	D-luciferin	Marcheva et al., 2010, Hou et al., 2019
*Bmal1 promoter*	Firefly luciferase	D-luciferin	Sadacca et al., 2011, Pulimeno et al., 2013
Non-alcoholic fatty liver disease			
*Albumin promoter*	Firefly luciferase	FFA- luciferin	Park et al. (2017)
		CCL-1	Heffern et al. (2016)
Obesity-mediated adipose inflammation			
*NF-κB promoter*	Firefly luciferase	D-luciferin	Fushiki et al. (2010)
*Saa3 promoter*	Firefly luciferase	D-luciferin	Sanada et al. (2016)
Thermogenesis of brown adipose tissue			
*Ucp1 promoter*	Firefly luciferase	D-luciferin	Galmozzi et al., 2014, Mao et al., 2017, Wang et al., 2019
*CIDEA promoter*	Luciferase 2	D-luciferin	Son et al. (2021)

RLuc, Renilla luciferase; YFP, yellow fluorescent protein; Bmp4 = Bone morphogenetic protein 4; Per = Period; BMAL1 = Basic helix-loop-helix ARNT, like 1; FFA, free fatty acid; CCL-1, Copper-caged luciferin-1; Saa3 = Serum amyloid A3; Ucp1 = Uncoupling protein 1; CIDEA, Cell death inducing DFFA, like effector a.

Leptin-deficient ob/ob (lep−/−) mice have been widely studied as an animal model of type 2 diabetes (Lindström, 2010). BLI for non-invasive monitoring of β-cell mass and function in ob/ob mice may provide new information on the regulation of β-cells in human type 2 diabetes. Patel et al. established the ob/ob-Luc mouse model using hybridization technology. The ability to monitor β cell function non-invasively in ob/ob mice provides new information for β cell regulation in type 2 diabetes (Patel et al., 2014).

MIP is not the only promoter used for BLI of β cells. *Rat insulin promoter* (RIP) is also widely used for pancreatic β-cell-specific transgene expression. Smith et al. generated RIP-Luc transgenic constructs using the RIP and luciferase gene and generated a RIP-Luc mouse model by DNA microinjection into FVB/N donor embryos (Smith et al., 2006). They found that glucose homeostasis and islet function were not significantly altered in RIP-Luc mice compared to those in wild-type FVB/N mice. However, luciferase expression was restricted to the islets of adult mice. Feeding adult RIP-Luc mice a high-fat diet resulted in enhanced bioluminescence signals. Since high-fat diets lead to insulin resistance in peripheral tissues (Muurling et al., 2002; Goldstein, 2003), researchers consider that the enhancement of the bioluminescence signal is related to the increase in the number of β cells or further activation of transgenic expression caused by metabolic changes in the body (Smith et al., 2006). However, the reason for this signal enhancement requires further study. Rats are commonly used as models for physiological research because of the advantages of their large size. However, owing to the relative lack of genetic models, the application of rats lags behind that of mice. Ghislain et al. was the first to apply BLI to rats and constructed RIP7-RLuc-YFP transgenic rats that specifically expressed RLuc-YFP fusion protein in pancreatic β cells (Ghislain et al., 2016).

Bone morphogenetic protein 4 (Bmp4) is a multifunctional growth factor mainly expressed in pancreatic β cells(Chen et al., 2004). Bmp4-Bmp receptor 1A signal transduction in β cells is necessary for insulin production and secretion. Mice with impaired signal transduction show impaired insulin secretion, leading to diabetes (Goulley et al., 2007; Scott et al., 2009). Yasunaga et al. constructed a Bmp4Luc reporter plasmid carrying the enhancer and promoter regions of the *Bmp4* and firefly luciferase genes. This construct was used to generate transgenic mice (p7kb-Bmp4-Luc) *via* pro-nuclear microinjection. After fasting for 24 h, the pancreas bioluminescence signal intensity of the transgenic mice increased approximately three-fold compared to that of the normal diet mice. Western blot analysis also showed that Bmp4 protein levels in the pancreases of mice after fasting increased approximately three-fold. This indicated that Bmp4 was altered in the starved state of mice, and this process could be reflected by the intensity of the BLI signal. The two reporter vectors p7kb-Bmp4Luc and pCMV-Luc were used to transfect cell lines. The BLI signal intensity of the cell lines with p7kb-Bmp4Luc was significantly increased after starvation medium treatment, and Western blot analysis also confirmed that the expression of Bmp4 was increased. These results suggest that Bmp4 is involved in physiological changes induced by starvation (Yasunaga et al., 2011).

Katsumata et al. used the *insulin I gene promoter* to construct a bacterial artificial chromosome (BAC) containing the luciferase gene. Ins1-Luc BAC transgenic mice were obtained by pro-nuclear injection of the chromosome into fertilized ICR mouse eggs. High-fat diet feeding and STZ induction confirmed that the bioluminescence signal intensity from islets changed with the increase and destruction of β cells. Researchers found that the BLI signal intensity produced by Ins1-Luc BAC transgenic mice was approximately four times that of MIP-Luc mice (Katsumata et al., 2013).

## 3 Application of BLI in circadian rhythm disorder

The circadian rhythm system is the primary regulator of human health and metabolism. It regulates metabolism by controlling body functions and synchronizing the peripheral clocks of almost all cells in the body. The suprachiasmatic nucleus (SCN) is the center which maintains circadian rhythms in the body. Period genes, including *Per1, Per2,* and *Per3*, are key components in the maintenance of central and peripheral circadian rhythms (Dunlap, 1999). Yamazaki et al. constructed a reporter fragment by linking the mouse *Per1* genome fragment with the firefly luciferase gene and injected it into fertilized eggs of Wistar rats to obtain Per1-Luc rats (Yamazaki et al., 2000). The SCN isolated from Per1-Luc rats maintained a circadian cycle similar to that of wild-type rats, indicating that the transgene did not alter the normal circadian rhythm. Moreover, the bioluminescence rhythm of SCN *in vitro* was detectable for up to 32 days. *In vitro* cultured liver, lung, and skeletal muscle tissues showed a circadian rhythm of BLI signals, but the circadian rhythm in peripheral tissues was attenuated after two to seven cycles of culture. Therefore, BLI has been gradually applied to study the circadian rhythm of central and peripheral tissues and organs.

The circadian rhythm of glucose tolerance and insulin sensitivity is important in human physiological functions (Poggiogalle et al., 2018). Previous studies have demonstrated that circadian rhythm disturbance in glucose metabolism is an important feature of type 2 diabetes (Mason et al., 2020). Marcheva et al. performed BLI of islets isolated from Per2-Luc transgenic mice (Marcheva et al., 2010). The BLI signal had a rhythmic variation with a period of 23.58 ± .3 h, which was very similar to the rhythm of SCN. The rhythm of islet BLI gradually weakened after 3 days, but the normal rhythm was restored after the addition of forskolin. Researchers have found that isolated islets with circadian rhythm disorders caused by clock gene mutations showed impaired glucose tolerance and decreased insulin secretion. Pancreatic islets have an autonomous circadian rhythm and play an important role in maintaining glucose homeostasis. Sadacc et al. used *basic helix-loop-helix ARNT like 1 (Bmal1)* Luc mice to demonstrate that the pancreas exhibits an autonomous circadian rhythm. The mRNA of insulin and clock genes (*Per1* and *Bmal1*) was co-expressed by immunohistochemical staining, which suggests that pancreatic β cells have a spontaneous circadian rhythm (Sadacca et al., 2011).

**FIGURE 2:**
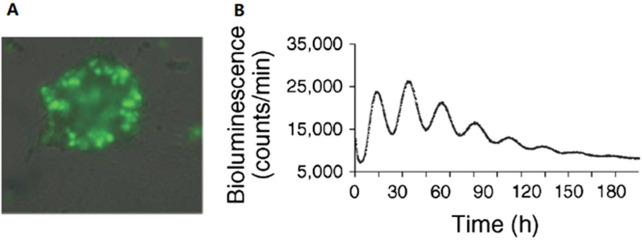
Autonomous circadian rhythm of human islets. **(A)** Luciferase and green fluorescent protein (GFP) are co-expressed in islet cells, and at least half of the cells in a single islet produce luciferase as demonstrated by the detection of GFP expression. **(B)** The expression of Bmal1-Luc had a distinct rhythm, with a cycle length of 23.6 ± .4 h, proving the existence of an autonomous circadian rhythm in human pancreatic islets. Reprinted (adapted) with permission from (Pulimeno et al., 2013).

In the mouse model, the circadian rhythm of the pancreas has been proven to play an important role in normal insulin release and glucose homeostasis, whereas mice with a disturbed pancreatic-islet circadian rhythm develop hypoinsulinemia and diabetes. An autonomous circadian rhythm in human pancreatic islets was first described by Pulimeno et al. (Figure 2). Researchers isolated and purified islets from organ donors and transduced human islets using a *Bmal1* luciferase lentivector. Using bioluminescence retardation microscopy, they recorded high-amplitude circadian oscillations at the islet population, individual islet, and scattered islet cell levels, indicating a functionally autonomous circadian rhythm in human islets (Pulimeno et al., 2013).

Previous studies have confirmed that diabetes leads to changes in the circadian rhythm of blood pressure, thereby increasing the incidence of cardiovascular disease (Ayala et al., 2013). Hou et al. established a new db/db-mPer2luc mouse model by crossing type 2 diabetic db/db mice with mPer2luc transgenic mice. The circadian rhythm of blood pressure in this mouse model was investigated by detecting rhythmic changes in bioluminescence signals both *in vivo* and *ex vivo*. Normal blood pressure was observed in db/db-mPer2luc mice; however, an impaired circadian rhythm of blood pressure was observed, which was associated with rhythm disturbances in baroreflex sensitivity, motor activity, and metabolism, but not heart rate or food and water intake (Hou et al., 2019).

## 4 Application of BLI in NAFLD

**FIGURE 3:**
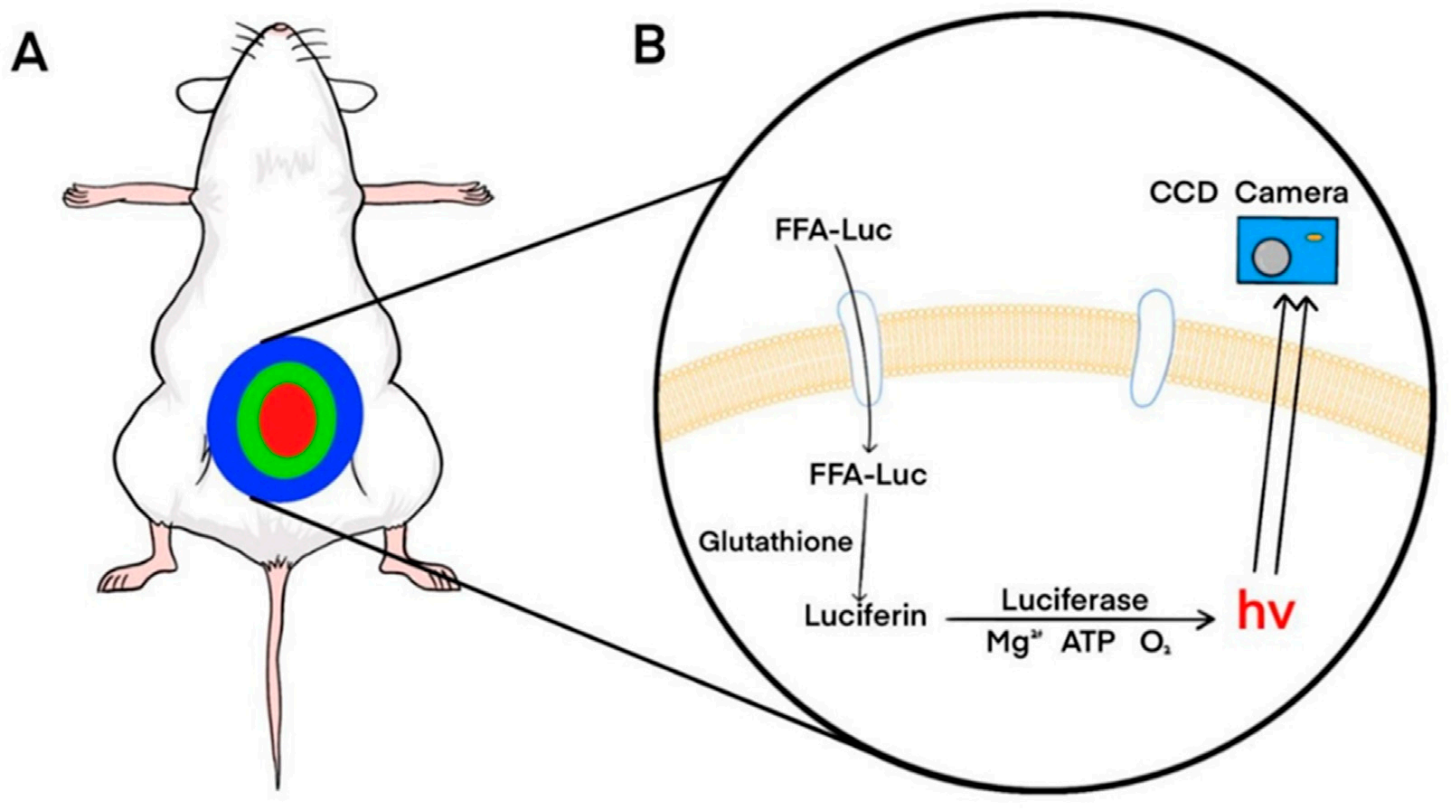
Principles of bioluminescence imaging of FFA-luc probes. **(A)** Bioluminescence imaging of transgenic mice expressing luciferase systemically or specifically in the liver after intraperitoneal injection of FFA-luc probes. **(B)** FFA-luc probes are transported into cells by transporters and reduced by intracellular glutathione to release free fluorescein. Luciferase catalyzes luciferin to produce photons that are detected by a charge-coupled device (CCD) camera. Reprinted (adapted) with permission from (Henkin et al., 2012). Copyright 2012 American Chemical Society.

Free fatty acid (FFA) uptake reflects the metabolic state of the liver and is closely associated with NAFLD (Fabbrini et al., 2010). To enable non-invasive monitoring of dynamic changes in FFA uptake, Henkin et al. developed an imaging probe based on long-chain fatty acids conjugated to luciferin (FFA-luc) (Henkin et al., 2012). Using BLI of mice expressing luciferase under the control of the actin promoter (FVB-luc+), FFA-luc accurately showed the changes in FFA uptake in the liver, adipose tissue, kidneys, and other organs (Figure 3). Park et al. generated transgenic mice (L-Luc mice) expressing luciferase under the control of an albumin promoter (Park et al., 2017). After intraperitoneal injection of FFA-luc, the bioluminescence signal from the L-Luc mice was liver-specific. Deoxycholic acid (DCA) can reduce the uptake of FFA-luc by inhibiting the activity of fatty acid transporters (FATPs), thereby reducing bioluminescence signal intensity(Nie et al., 2012). In contrast, Henkin et al. found fenofibrate enhanced FFA uptake, thereby enhancing signal strength. They have also found a strong circadian rhythm in the uptake of FFAs by the liver. The development of applications for non-invasive monitoring of FFA uptake will help improve our understanding of liver lipid metabolism, and this technology can be applied to other lipids, such as triglycerides and cholesterol.

Copper (Cu) is an essential element in humans and other mammals. Studies have reported that copper metabolism imbalance is related to metabolic disorders, such as obesity, diabetes, and NAFLD (Lutsenko et al., 2007). Therefore, longitudinal monitoring of changes in Cu content is important for the study of changes in Cu metabolism and related physiological and pathological processes. Heffern et al. developed Copper-Caged Luciferin-1 (CCL-1), a bioluminescence-based probe that reflects changes in Cu levels *in vivo* with high sensitivity and selectivity (Heffern et al., 2016). Using CCL-1, researchers measured hepatic Cu levels in mice with high-fat-induced NAFLD. First, they validated the function of CCL-1 in FVB Luc mice, which express luciferase in most organs. CCL-1 can accurately reflect changes in Cu levels in FVB Luc mice after treatment with CuCl_2_ or the Cu-chelating agent ATN-224. However, D-fluorescein could not reflect the fluctuation in Cu level. Next, the researchers performed BLI of L-Luc mice fed a high-fat diet and normal diet. The bioluminescence signal intensity of CCL-1 decreased significantly with time in the early stage of high-fat feeding, but this decline was not observed in mice fed a normal diet. This indicates that Cu deficiency occurs before the occurrence of NAFLD, and may even promote the occurrence of NAFLD. Western blot analysis of liver tissue from high-fat-diet mice revealed increased expression of the Cu export proteins ATP7A and ATP7B compared with that of mice fed a normal diet. This suggests that, in a high-fat diet-fed NAFLD model, Cu deficiency may be due to increased hepatic Cu output.

In 2018, this team synthesized the N-acetylgalactosamine-functionalized ionophore, which can deliver copper to the liver to replenish the copper pools (Su et al., 2018). After adding the hepatic copper delivery agent to FVB-Luc mice, the bioluminescence signal intensity of CCL-1 in the liver was significantly increased, which verifies this agent can supplement the lack of copper to the liver without causing increased copper levels in other organs.

## 5 Application of BLI in obesity-mediated adipose inflammation

Low-grade inflammation of adipose tissue is associated with obesity and contributes to the development of obesity-related diseases such as insulin resistance, hypertension, and arteriosclerosis (Hotamisligil, 2006). Nuclear factor-kappa B (NF-κB) is a key regulator of various inflammatory responses (Barnes and Karin, 1997). Previous studies have shown that NF-κB transcript levels are elevated in the adipose tissue of patients with obesity (Weisberg et al., 2003). The activation of NF-κB further aggravates the inflammation of adipose tissue and promotes the proliferation of adipocytes (Catrysse and van Loo, 2017). Fushiki et al. successfully monitored the expression of NF-κB in adipose tissue using BLI (Fushiki et al., 2010). They established NF-κB-mediated luciferase-expressing 3T3L1 (3T3-L1/NF-κB-re-luc2P) cells. Both tumor necrosis factor-α (TNF-α) and macrophage stimulation significantly increased the intensity of the bioluminescence signals produced by 3T3-L1/NF-κB-re-luc2P adipocytes. *In vitro* experiments demonstrated that this cell line could reflect the inflammatory response through changes in the bioluminescence signal intensity. The researchers implanted equal numbers of 3T3-L1/NF-jB-re-luc2P adipocytes into the epididymal white adipose tissue (eWAT) of diet-induced obesity (DIO) mice and lean mice. There was no difference in bioluminescence signal intensity early post-implantation; however, DIO mice showed an enhanced bioluminescence signal several hours later and maintained more than 20 times the bioluminescence signal of lean mice for 3 weeks. Real-time RT-PCR analysis also demonstrated activation of NF-κB in the eWAT of DIO mice. This model successfully non-invasively monitored the relationship between obesity and adipose inflammation in real time.

Macrophage infiltration plays an important role in adipose-tissue inflammation and metabolic disorders (Thomas and Apovian, 2017). Recent studies have shown that the number of macrophages in adipose tissue correlates with the degree of obesity and that weight loss induced by surgery or exercise leads to a reduction in the number of macrophages in the adipose tissue of obese mice (Kosteli et al., 2010; Thomas and Apovian, 2017). Therefore, monitoring the number of macrophages in adipose tissue is critical for assessing the obesity-related adipoinflammatory status. Sanada et al. found that the expression level of the adipocyte-derived *serum amyloid A3* (Saa3) gene was affected by macrophages infiltrating the adipose tissue (Sanada et al., 2016). Therefore, transgenic mice (Saa3-Luc mice) were constructed using the Saa3 promoter. *In vivo* BLI of high-fat diet-fed Saa3-Luc mice revealed that the signal intensity was much higher than that of conventional diet-fed Saa3-Luc mice. Western blot analysis of white adipose tissure from Saa3-Luc mice shows that high-fat diet-induced obesity increases the expression level of Saa3-driven luciferase. These evidences demonstrate the massive infiltration of macrophages in the adipose tissue of obese mice (Sanada et al., 2016).

## 6 Application of BLI in thermogenesis of brown adipose tissue

Brown adipose tissue (BAT) plays a key role in mammalian metabolism and thermogenesis (Cannon and Nedergaard, 2004). It converts energy into heat through non-shivering thermogenesis, and thus has great therapeutic potential for several metabolic disorders, such as obesity and diabetes (Tan et al., 2011). Many studies have shown that mesenchymal stem cells (MSCs) are suitable candidates for the formation of new BAT (Neubauer et al., 2004; Alhadlaq et al., 2005; Mauney et al., 2005; Rim et al., 2005). However, in these studies, adipogenesis was induced by adding adipogenesis supplements to the growth media, and MSCs needed to be cultured in the media for a long time to achieve adipogenesis. Peroxisome proliferator-activated receptor γ (PPARγ2) and CCAAT/enhancer-binding protein alpha (C/ebpα) are nucleic acid receptors that mediate brown adipogenesis (Kang et al., 2005; Nedergaard et al., 2005). Sheyn et al. stated that transfection of PPARγ2 and C/ebpα into MSCs can induce rapid differentiation into adipocytes, and genetically modified stem cells can be directly implanted into the site of interest to complete the differentiation process *in vivo*. Using nuclear infection technology, PPARγ2 and C/ebpα were overexpressed in MSCs carrying the luciferase gene. After the engineered cells were implanted into mice, new adipose tissue was detected. BLI and immunostaining indicated that the new adipocytes expressed luciferase and the BAT marker, uncoupling protein 1 (UCP1) (Sheyn et al., 2013).

BAT can fight obesity by releasing energy as heat through UCP1 (Kopecky et al., 1995). Therefore, Galmozzi et al. developed *Ucp1*-luciferase mice. Luciferase activity in the model body reliably simulated the expression of endogenous UCP1 and its response to physiological stimuli. Chemical screening of brown adipose cells derived from this model revealed a compound (WWL113) that can enhance the expression of UCP1 *in vivo* and promote thermogenesis of BAT (Galmozzi et al., 2014). Notably, three-dimensional (3D) reconstructions were calculated by two-dimensional (2D) bioluminescence signals using a specific algorithm in this study. 3D imaging makes up for the defect that 2D imaging can only achieve relative positioning and quantification of bioluminescence signals. The latest generation of equipment, IVIS Spectrum, can scan bioluminescence signals by tomography and conduct 3D reconstruction of imaging results through a model algorithm. The 3D information of bioluminescence signal *in vivo*, such as depth, luminescent volume, luminescent intensity, cell number, and probe concentration, can be more easily obtained (Luli et al., 2016).

However, the luciferase reporter driven by the *Ucp1* promoter constructed by Galmozzi et al. was integrated into the Y chromosome, which restricted the model to be only applicable to male mice. Mao et al. constructed *Ucp1*-2A-Luciferase knock-in Mice (Mao et al., 2017). They replaced the endogenous *Ucp1* termination codon with the firefly luciferase coding sequence so that luciferase and UCP-1 could be co-expressed. This method relieves the influence of mouse sex on UCP-1 and luciferase production. Wang et al. constructed a transgenic mouse model called *Ucp1* dual-reporter-gene mouse (Wang et al., 2019). This model expresses firefly luciferase and near-infrared red fluorescent protein (iRFP713) driven by endogenous *Ucp1* gene regulatory elements.

Cell death-inducing DNA fragmentation factor-like effector A (CIDEA) is a lipid droplet-associated protein (Barneda et al., 2015). Recent studies have confirmed that the expression level of CIDEA reflects the thermogenic capacity of BAT (Jash et al., 2019). For non-invasive monitoring of CIDEA expression levels, Son et al. established CIDEA reporter mice expressing CIDEA, luciferase 2 (Luc2), and tandem-dimer tomato (tdT) proteins under the control of the *Cidea* promoter. This mouse model was used to reveal that luciferase activity and bioluminescence signal levels were positively correlated with thermogenic gene expression and mitochondrial oxidative metabolism(Son et al., 2021).

## 7 Conclusion

This review describes the increasing applications of BLI models in the field of MetS research. Researchers have gained information about physiological processes *in vivo* by observing increasing, decreasing, or rhythmic changes in the intensity of bioluminescent signals. BLI provides a unique aid for the mechanistic and therapeutic exploration of MetS-related diseases. Undeniably, all the BLI models mentioned above have their unique advantages. *Mouse insulin promoter* (MIP) and *rat insulin promoter* (RIP) are the most widely used promoters in diabetes and β cell regeneration research(Smith et al., 2006; Yin et al., 2013). MIP-Luc mice and RIP-Luc mice have also been widely commercialized. Researchers increased the expression level of luciferase through transgenic technology. Bacterial artificial chromosome (BAC) transgenesis was applied in Katsumata’s research. The BLI models generate stronger bioluminescence signals because BAC contains almost all regulatory sequences necessary for the expression of luciferase (Son et al., 2021). In the study of NAFLD, researchers designed two different fluorescein substrates (FFA-luc and CCL-1) to meet the needs of the research purpose (Henkin et al., 2012; Heffern et al., 2016). Son et al. chose luciferase 2 as the luciferase gene to study the role of CIDEA in thermogenesis of brown adipose tissue(Son et al., 2021). Luciferase 2 can transmit more than four times light emission by optimizing codons and removing transcription factor binding sites, which is more than 100 times stronger than the bioluminescence signal generated by luciferase (Gil et al., 2012).

BLI has many advantages, including low levels of interfering signals, high sensitivity, and relatively low cost (O'Neill et al., 2010; Mezzanotte et al., 2017). Furthermore, BLI permits multiple non-invasive physiological process monitoring in animals, without the need for euthanasia (Syed and Anderson, 2021). These advantages make BLI a valuable tool for studying the spatial and temporal dynamics of MetS in living organisms. BLI has flaws and shortcomings, such as low resolution, poor tissue penetration, and the necessity of gene editing. With further development of BLI, these shortcomings are gradually being overcome. Traditional BLI applications can monitor as little as 50 cells. A study in 2018 showed that researchers established AkaBLI, an all-engineered bioluminescence *in vivo* imaging system. The light produced by AkaBLI in the body is 100–1,000 times brighter than traditional systems. With the aid of AKaBLI, bioluminescence signals of individual tumor cells in experimental animals can be monitored(Iwano et al., 2018). Bioluminescence resonance energy transfer (BRET) can transfer the resonance energy of luciferase to a neighboring fluorescent protein, which emits higher wavelength light. Based on the principle of BRET, the researchers developed suitable luciferases and fluorescent proteins to improve the sensitivity and tissue penetration of the BLI system(Dragulescu-Andrasi et al., 2011; Endo and Ozawa, 2020). BLI based on BRET can also monitor protein-protein interactions in small animals, which has great potential for pharmacological and physiological research(Saito et al., 2012). The development of multimodal imaging techniques in recent years can overcome the shortcomings of single-modal imaging techniques. As optical imaging techniques, BLI and fluorescence have the advantages of cost-effectiveness, and BLI can be used for non-invasive real-time imaging. PET can provide tomographic images and locate deep tissues. The combination of these imaging techniques can play respective advantages, more precisely identify the location and physiological processes of specific cells and their interactions with neighboring cells (Doubrovin et al., 2004; Yong et al., 2011).

The photons produced by bioluminescence are absorbed by tissue, which affects the accuracy of BLI and limits the application of this technique in large animals. Editing the luciferase reporter to enhance the transcriptional level of luciferase or modifying the luciferin substrate to produce longer-wavelength light for stronger tissue penetration may enable the application of BLI to deeper tissues or larger animals(Lu et al., 2020). BLI has been used as a preclinical technology in medical research, but it still requires challenging work until it can be applied to the human body. The premise of BLI is to genetically edit organisms to express luciferase; however, gene editing in humans is currently impractical. Humans cannot express luciferase through gene editing; however, lipid nanoparticles have been tested in animal models for mRNA delivery (Hajj et al., 2020). Luciferase mRNA is encapsulated in lipid nanoparticles and transported into target cells, resulting in luciferase expression *in vivo*. This technology will provide theoretical support for the BLI of target organs or certain cancer tissues in the human body. BLI may be used as a clinical technology for the diagnosis and treatment of MetS and other diseases in humans in the near future.
